# International Congress on Tuberculosis

**Published:** 1904-01

**Authors:** 


INTERNATIONAL CONGRESS ON TUBERCULOSIS.—The Committee on Organization of the International Congress on Tuberculosis appointed by the Hon. David R. Francis, President of the Universal Exposition, St. Louis, 1904, met at the Press club in the city of York on the 11th day of November, 1903, and took the preliminary steps to organize an International Congress on Tuberculosis.

Nearly every member of the committee was present or represented by proxy. It was unanimously resolved to organize an International Congress under the following name: "American International Congress on Tuberculosis, to be held October 3d, 4th, and 5th, 1904, under the auspices of the Universal Exposition, St. Louis, 1904, and of the American Congress on Tuberculosis."

The present officers of the American Congress on Tuberculosis were, with a few changes and exceptions, elected to the corresponding offices in the American International Congress on Tuberculosis.

It was unanimously resolved that the Congress be open to the legal, medical, and all other professions, judges, philanthropists, scientists, statesmen, legislators, and all citizens interested in averting the ravages of the dread disease, and that they be invited to cooperate and become members of the body. * * *

The chairman of the committee on organization laid before the committee a communication from Howard J. Rogers, director of congresses, informing the committee that Surgeon-General Nicholas Senn of Chicago, Illinois, and Dr. Thomas Darlington of New York, had been added to the committee on organization.—*Extract from Medico-Legal Journal Advance Sheets.*

[Dr. Darlington is now president of the Board of Health of the City of New York.—EDITOR.]

---

"ANTIPHLOGISTINE renders ready service to the patient and physician by promptness and positiveness of action."

